# CRISPR/Cas9-Mediated Customizing Strategies for Adoptive T-Cell Therapy

**DOI:** 10.3390/pharmaceutics16030346

**Published:** 2024-03-01

**Authors:** Hyeseon Park, Yoo Kyung Kang, Gayong Shim

**Affiliations:** 1 School of Systems Biomedical Science and Integrative Institute of Basic Sciences, Soongsil University, Seoul 06978, Republic of Korea; ritapark@soongsil.ac.kr; 2 College of Pharmacy and Research Institute of Pharmaceutical Sciences, Gyeongsang National University, Jinju 52828, Republic of Korea

**Keywords:** CRISPR/Cas9, cancer immunotherapy, immune checkpoint, T-cell therapy

## Abstract

Clustered regularly interspaced short palindromic repeat-associated protein Cas9 (CRISPR/Cas9) technology is at the forefront of cancer immunotherapy innovation, offering precise and personalized treatment strategies. In this review, we discuss CRISPR/Cas9’s ability to precisely edit the genome, its impact on immune checkpoint control, and its application in immune cell engineering, where it surpasses traditional gene editing techniques. Originally inspired by bacterial defense mechanisms, this technology has made great strides in cancer immunotherapy as a mechanism to specifically target the PD-1/PD-L1 pathway in immune checkpoint blockades. In addition, CRISPR/Cas9 plays an important role in cancer treatment by facilitating genetic modifications to enhance the properties of adoptive cell therapy, optimizing the therapeutic potential of this approach. This review provides an overview of the development of CRISPR/Cas9, its important role in immune checkpoint control, applications in immune cell engineering, and the current status of clinical trials. However, safety concerns related to off-target effects and unintended mutations require continued research and caution. Continued advances in CRISPR technology hold the promise of revolutionizing the cancer treatment paradigm, providing personalized and effective therapies for patients with various types of cancer.

## 1. Introduction

Clustered regularly interspaced short palindromic repeats (CRISPRs), a groundbreaking technology, have caused remarkable advancements in genetic engineering [1,2]. This powerful tool, often paired with the CRISPR-associated protein Cas9 (CRISPR/Cas9), is the gold standard for genome editing in several mammalian cells, surpassing earlier gene editing techniques such as zinc-finger nucleases and transcription activator-like effector nucleases [3,4,5]. CRISPR precisely manipulates the target genetic sequences through the guidance of the Cas9 endonuclease by a single-guide RNA (sgRNA), allowing the introduction of desired changes, such as gene insertions or deletions, with the utmost accuracy [2,4,6]. The simplicity and effectiveness of this technique have sparked a revolution in genetic research and have profound implications in medical science [5,7,8].

In recent years, CRISPR technology has been widely used in cancer treatment, specifically in immunotherapy and cell-based therapies [9,10,11]. This approach harnesses the patient’s immune system to combat cancer with unprecedented precision and potency [9,12]. CRISPR-based cancer immunotherapies have garnered significant attention and hold immense promise as a new frontier in the battle against cancer [9,11]. CRISPR-based cancer immunotherapy targets the immune checkpoint pathway in cancer cells to regulate immune cell activation, proliferation, and survival, enhancing the release of cytotoxic substances into the tumor microenvironment and improving resistance to tumor chemotherapy [13,14,15,16,17,18,19]. Additionally, a crucial aspect involves modifying specialized immune T cells using CRISPR technology [9,20,21].

Several innovative and personalized cancer treatment strategies have been developed using the precision and versatility of CRISPR, potentially overcoming one of cancer’s most notorious strategies for evading the immune system [22,23,24]. As research continues to advance, the possibilities of CRISPR-based cancer therapies seem boundless, providing renewed hope to cancer patients and their families worldwide [11,22,25,26].

## 2. CRISPR/Cas9 Advances: From Bacterial Defense to Genetic Correction

The CRISPR/Cas9 system is a remarkable tool that mimics the adaptive immune system in bacteria, primarily serving as a defense mechanism against bacteriophages [1,2,27]. It operates through a well-defined three-stage sequence: acquisition, transcription, and interference. This process begins with the acquisition of foreign DNA, which is seamlessly integrated into the host CRISPR locus [28,29]. Subsequently, the mature CRISPR-derived RNA (crRNA) is transcribed and the Cas protein directly cleaves the target DNA sequence under the guidance of RNA [30].

In particular, the CRISPR/Cas system comprises a single Cas9 endonuclease and guide RNA (gRNA) when employing type II *Streptococcus pyogenes* Cas9. This gRNA typically consists of a 20-nucleotide-long crRNA, which engages in complementary base pairing with DNA target sequences, along with transactivating CRISPR RNA (tracrRNA), forming a complex with Cas9 to facilitate precise site-specific DNA double-strand breaks (DSBs) [31]. This tracrRNA and crRNA combination can be conveniently designed as a single RNA molecule, known as sgRNA, which greatly enhances its practicality and versatility [32]. To elucidate, sgRNA, guided by this single RNA entity, accurately recognizes specific DNA target sequences, while the Cas9 endonuclease induces a DSB in the target sequence, typically positioned around three base pairs upstream of the protospacer adjacent motif (PAM) sequence [33]. Notably, Cas9 cannot entirely recognize complementary target sequences in the absence of PAM [34]. Thus, PAM is a critical prerequisite for sgRNA design and plays a pivotal role in both the adaptation and interference stages of the CRISPR system [35]. Moreover, the system’s simplicity facilitates the design of multiple sgRNAs, enabling multiple simultaneous edits at the target locus [36].

After DSB induction, cells initiate gene repair processes through their intrinsic self-repair mechanisms involving non-homologous end joining (NHEJ) or homology-directed repair (HDR) [37,38,39,40]. NHEJ typically results in insertions or deletions (INDELs) within genes, causing disruptions in protein-coding sequences and ultimately yielding functional knock-outs [38]. Conversely, HDR, which employs donor DNA templates, allows precise gene insertion at CRISPR cleavage sites or accurate gene correction with specific sequences [39]. Based on this, the application of genetic correction strategies opens various possibilities across different scientific fields, including novel animal model creation, drug development, genomic surgery, and live imaging [23,24,41]. This technology, characterized by the high precision and efficiency in genetic manipulation, presents innovative potential that distinguishes it from conventional research methods.

**Figure 1:**
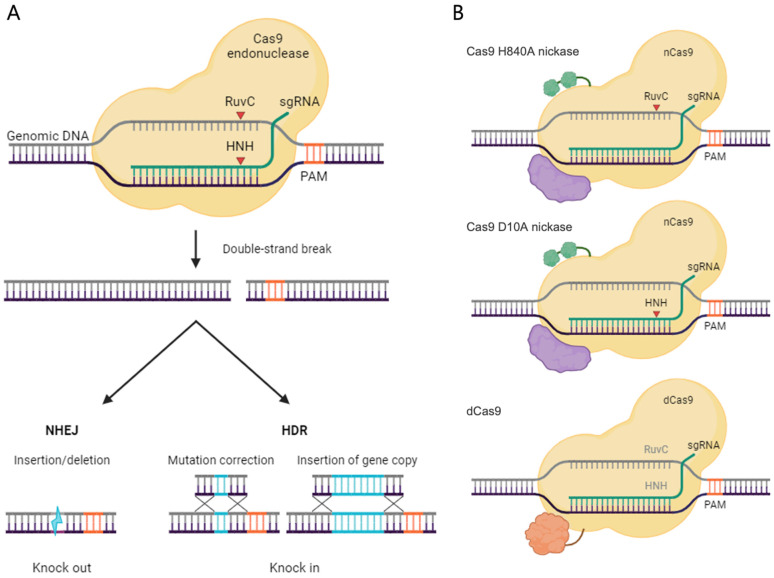
An overview of clustered regularly interspaced short palindromic repeat-associated protein Cas9 (CRISPR/Cas9) gene editing and its variants. (**A**). CRISPR/Cas9-mediated genome editing: The lone Cas9 endonuclease, when combined with sgRNA, attaches to the target DNA and causes double-strand breaks (DSBs) in three bases upstream of the protospacer adjacent motif (PAM) sequence. In mammalian cells, DSBs are repaired via non-homologous end joining (NHEJ) or homology-directed repair (HDR). After CRISPR/Cas9 intervention, NHEJ facilitates gene knock-out and HDR enables gene knock-in. (**B**). Cas9 endonuclease amino acid residues H840 and D10 are located in the HNH and RuvC domains, respectively. Cas9 D10A only cleaves strands that bind complementarily to gRNA, while Cas9 H840A only cleaves non-target strands. dCas9 has both D10A and H840A mutations, allowing it to bind to DNA targets without cleavage. To regulate gene expression, dCas9 sometimes pairs with effector proteins such as KRAB and VPR. Deaminases or reverse transcriptases are at times combined with nCas9 for precise gene editing. Created with BioRender.com (accessed on 19 January 2024).

Furthermore, a profound understanding of the molecular structure of *Streptococcus pyogenes* Cas9 has paved the way for refining and expanding the mechanisms of DNA DSB generation [42]. This has developed dCas9 (dead Cas9) and nCas9 (Cas9 nickase), among others, which have been applied to various genetic correction projects (Figure 1). In the expanded application of dCas9-based CRISPR, there are mechanisms such as CRISPR activation (CRISPRa), where effector proteins binding to dCas9 regulate gene expression, activating it. Conversely, CRISPR interference (CRISPRi) induces a mechanism to inhibit gene expression. In CRISPRi and CRISPRa, a drawback was the need for continuous expression of artificial proteins for cells to maintain the changes. Researchers have addressed this limitation by developing a new technology called CRISPRoff, which serves as an on–off switch for gene editing. It involves the transient expression of the CRISPRoff protein, leading to the creation of an epigenetic program that cells maintain through division. Notably, CRISPRoff can silence genes, even those without large, methylated regions known as CpG islands. Since this method does not alter the DNA sequence, researchers can reverse the silencing effect using enzymes to remove methyl groups, referred to as CRISPRon. The fusion of nCas9 with deaminases enhances its efficiency, serving as a precise base editor for gene correction. In this regard, two types of base editors (BEs), namely Cytosine Base Editors (CBEs) and Adenine Base Editors (ABEs), have been reported in the context of dCas9 utilization. Furthermore, the utilization of reverse transcriptases as effector proteins expands the scope beyond conventional DNA editing, encompassing not only insertions and deletions (indels) but also small genomic alterations. These modified Cas9 variants not only enable precise gene editing while minimizing off-target effects, but also open new possibilities for applications such as cancer immunotherapy [42,43,44,45,46,47].

## 3. CRISPR/Cas9 Technology Revolutionizing Immune Checkpoint Control in Cancer Cells

The cancer microenvironment manifests in various aspects, including interactions between cancer cells and surrounding tissues, cellular diversity, vascular supply, and the influence of the immune system [48,49]. Cancer can be categorized into various subtypes, each with unique characteristics attributed to the diversity within the tumor microenvironment [50,51]. Some tumors may develop resistance to specific treatments, which often stems from the tumor microenvironment diversity. The diversity in the microenvironment can hinder or modify the functionality of immune cells within the tumor, leading to cancer evading detection or immune responses [52,53,54].

This complexity poses challenges to conventional cancer treatment methods and frequently results in drug resistance, recurrence, and unfavorable prognosis, thereby compromising the effectiveness of traditional approaches [54,55]. In response to these challenges, cancer treatment regimens, particularly focusing on immune checkpoint inhibitors (ICIs) [56,57], have been developed. ICIs activate the immune system to effectively combat cancer and overcome immune evasion [58,59]. Moreover, the design of ICIs can be tailored to specifically target certain subtypes, promote efficient immune cell activation, and provide personalized treatment strategies for various tumors that exhibit robust immune responses [14,60,61].

Using CRISPR technology, researchers are exploring innovative ways to modulate the programmed cell death protein 1/programmed cell death protein ligand 1 (PD-1/PD-L1) immune checkpoint control pathway, which holds promise for overcoming one of the most notorious strategies for evading the immune system. In cancer immunotherapy, the capabilities of CRISPR/Cas9 extend to the tumor microenvironment, and one of the main approaches in CRISPR-based cancer immunotherapy is to target the PD-1/PD-L1 pathway in cancer cells [19,62]. Immune checkpoints, such as PD-1/PD-L1, play a pivotal role in regulating the immune response in cancerous tissues [11,63]. Typically, the interaction between PD-1 and PD-L1 leads to immune suppression by inhibiting T-cell function and promoting immune evasion in PD-L1-expressing tumor cells [64,65]. Therefore, the precise targeting and removal of PD-L1 from tumor cells using CRISPR/Cas9 is important for fine-tuning cancer immunotherapy. This mechanism significantly reduces PD-L1 expression within tumor cells, significantly improving the complex tumor microenvironment [66,67]. PD-L1 inhibition within the tumor microenvironment activates the immune cells through several mechanisms, including T-cell proliferation. Specifically, this manifests as an increase in immune supporters, such as CD4 T cells, CD8 T cells, NK cells, and CD11c M1 macrophages. They also promote anti-cancer immune responses by suppressing regulatory T cells [16,17,18]. This remarkable genetic alteration also notably changed cytokine expression profiles within the tumor microenvironment. This change promotes the production of inflammatory cytokines, which play an important role in enhancing anti-cancer immune responses [16,18,68]. However, CRISPR/Cas9 also enhances the sensitivity of cancer cells to chemotherapy, modulates their ability to release cytotoxic substances through precise PD-L1 targeting, and improves their resistance to tumor chemotherapy, as well as amplifies the therapeutic effect of chemoradiotherapy. This revolutionary technology has been instrumental in countering chemotherapy resistance, which is a major barrier to cancer treatment [16,18,69]. Its applications span a broad spectrum, including melanoma, breast cancer, osteosarcoma, and several other types of cancer [17,69,70,71,72].

Unlike stimulating T-cell activity, the CD47–Signal regulatory protein alpha (SIRPα) axis functions as a regulatory point for tumor phagocytosis. This axis serves as a myeloid-specific immune checkpoint, impacting the innate immune system. The presence of CD47 on tumor cells hinders macrophages in the immune system from undergoing phagocytosis, and noteworthy outcomes have been documented in clinical trials that focus on CD47 in various blood cancers and solid tumors. Furthermore, the effectiveness of the CD47-SIRPα signal relies on the phagocytic capabilities of macrophages, the predominant infiltrating cells in tumors, suggesting that directing attention to CD47 marks a pivotal development in cancer immunotherapy.

Various treatment strategies, including the use of antibodies targeting CD47, RNA interference for *CD47* silencing, and agents modulating CD47 expression, have demonstrated efficacy in cancer treatment by disrupting the interaction between CD47 and SIRPα. However, these approaches may show temporary effects and might not ensure a sustained anti-tumor immune response in the long term. Clinical trial results indicate that anti-CD47 monotherapy is ineffective against solid tumors, particularly in immunocompetent mice with poorly immunogenic tumors or immunodeficient mice with highly immunogenic tumors. Even when tumor cells are opsonized with an ineffective monoclonal antibody (anti-Tyrp1) and enhanced through CRISPR interference (CRISPRi), poorly immunogenic solid tumors remain challenging. However, a relatively uniform minimum of 80% CD47 repression achieved through CRISPR interference (CRISPRi), combined with the ineffective monoclonal antibody (anti-Tyrp1), enables phagocytosis to dominate net growth, triggering an effective anti-tumor immune response [73].

Furthermore, studies using the CRISPR/Cas9 delivery system have reported tumor-specific *CD47* inhibition and, additionally, the transformation of tumor cells into IL-12 production factories. This suggests that the combination of tumor-specific *CD47* inhibition and IL-12 production can synergistically enhance macrophage-mediated immunotherapy [74]. In summary, CRISPR/Cas9 appears to be a versatile and powerful strategy for inducing profound changes within the tumor microenvironment through the strategic manipulation of the PD-1/PD-L1 pathway to induce the genetic modulation of immune cells and amplify the effectiveness of cancer therapies, thus emerging as a potent weapon against chemotherapy resistance.

## 4. CRISPR/Cas9-Enhanced Immune Cell Engineering

**Figure 2:**
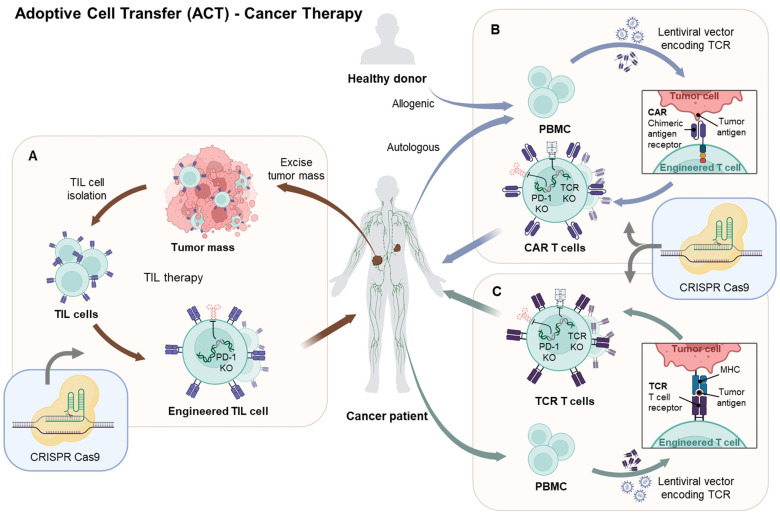
Advancing adoptive cell therapy: a comprehensive exploration of CRISPR/Cas9-mediated enhancements. CRISPR/Cas9 is used to enhance the therapeutic capabilities of adoptive cell therapy. (**A**) In tumor-infiltrating lymphocytes (TILs), the use of CRISPR has focused on removing PD-1 or *CISH* to accentuate a sustained response. (**B**) In allogeneic CAR-Ts, genetic modification of the native TCR and MHC-I reduces the risk of graft-versus-host disease and host-versus-transplant reactions, enabling off-the-shelf CAR-T-cell therapy and mitigating T-cell exhaustion when killing targets such as PD-1. (**C**) TCR-Ts can also be functionalized and engineered with CRISPR to increase the specificity and effectiveness of therapy. Created with BioRender.com (accessed on 5 February 2024).

Recent advancements in immunotherapy have proven effective in treating various types of cancers, particularly through adoptive cell therapies (ACTs) involving tumor-infiltrating lymphocytes (TILs) or genetically modified cells like transgenic T-cell receptor (TCR) lymphocytes or CAR-T cells [75]. However, challenges such as cancer cells evading immune surveillance and complexities within the tumor microenvironment hinder the long-term success of immunotherapy. This has led to the exploration of innovative strategies to understand and enhance the overall effectiveness of immunotherapy. In addressing these challenges, CRISPR technology plays a crucial role by precisely targeting genes specific to these activities (Figure 2).

TIL therapy, an early form of ACT, entails extracting lymphocytes from a tumor, cultivating and expanding them in vitro, and subsequently reintroducing them into the patient. Research is ongoing to assess the effectiveness of cancer therapy, particularly by eliminating PD-1 or *CISH* to emphasize the sustained response of TILs. However, challenges in isolating and expanding TILs, coupled with the modest anti-cancer effects of this approach, have led to the development of engineered TCR-T or CAR-T cells [76].

Among these, CAR-T employs antigen recognition sites on antibodies to detect cancer cells, specifically recognizing antigens expressed on the “cancer cell surface.” However, it has limitations, as cancer cells can evade CAR-T attacks by mutating target molecules. Therefore, the application of CRISPR is instrumental in optimizing CAR-T-cell production and ensuring they are finely tuned for their therapeutic roles [77,78]. This gene editing process involves various crucial genes, including TCRs, the major histocompatibility complex (MHC), and PD-1, which have significant involvement in immune responses and cancer immunotherapy [13,75,79].

Central to this genetic modification was the removal of *TRAC*, which encodes the TCR constant alpha chain, an integral component of the TCR complex [80]. CRISPR aims to reduce the presence of TCR molecules on engineered T-cell surfaces by strategically deactivating *TRAC*. This reduction both minimizes graft-versus-host disease (GvHD) risk and optimizes the functionality of these modified T cells for their therapeutic roles [81,82,83]. In addition to *TRAC*, CRISPR focuses on genes like β2-Microglobulin (*B2M*), a crucial component of the MHC class I molecule. *B2M* deactivation plays a pivotal role in downregulating inhibitory signals and ICIs, ultimately enhancing the cytotoxicity and anti-tumor capabilities of CAR-T cells [14,15]. Moreover, CRISPR-based gene editing has expanded the horizons of cancer immunotherapy to the immune checkpoint protein PD-1, akin to the modulation of the PD-1/PD-L1 pathway by PD-L1 inhibition in cancer cells. Using CRISPR, researchers can effectively deactivate PD-1, thereby alleviating its inhibitory effects [9,77]. This strategic PD-1 deactivation unleashes the complete potential of immune cells, leading to an elevation in crucial cytokines such as interferon gamma (IFN-γ) and interleukin-2 (IL-2). The heightened secretion of these cytokines fortifies the immune response against cancer cells, thereby augmenting the efficacy of treatment [19]. CRISPR technology, however, does not stop at gene deactivation; it takes a step further by selectively eliminating genes like transforming growth factor beta (*TGF-β*) and prostaglandin E2 (*PGE2*), both known for their immunosuppressive properties. Removing these genes not only minimizes potential toxicities but also creates a more favorable microenvironment for engineered immune cells to function optimally [84,85].

To further enhance the immune response, CRISPR can be employed to introduce genes such as *IFN-γ* and *IL-2*, which are well-known for their roles in enhancing cytokine production. This increase in cytokine secretion may provide CAR-T cells with an additional advantage in the battle against cancer. Conventional CAR-T-cell production is hindered by high costs and time-consuming autologous T-cell production, potentially leading to disease progression in some patients [86,87]. Developing universal allogeneic T cells from healthy donors that are resistant to immunosuppressive molecules and involve the removal of TCR, *MHC-1*, and *HLA-1* genes using gene editing tools, such as CRISPR/Cas9, while simultaneously disabling PD-1, is crucial to overcome these limitations [15,83,88]. Universal CAR-T cells offer immediate availability, increased efficacy, consistency, enhanced accessibility, and flexible dosing [82,89].

In particular, as the specificity of cancer antigens has increased and it has become technically feasible to clone the TCR, the T-cell receptor that responds to cancer antigens, it has become possible to develop TCR-T therapies that produce large numbers of T cells that recognize cancer cells. In addition, the use of TCR is generally associated with a lower frequency of cytokine release syndrome [90]. Methods are also being developed to modulate T-cell receptors (TCRs) using CRISPR/Cas9 non-viral precision gene editing. Target-specific TCRs can likewise be isolated and modified from healthy donors, and in clinical trials, TCR genes are simultaneously modified in patients’ T cells to enhance existing autoimmune responses and effectively target cancer. Enhanced, genetically engineered T cells were detected in tumor biopsy samples after infusion at a higher frequency than endogenous TCRs before infusion [22].

In summary, CRISPR technology serves as a versatile molecular tool that enables the precise genetic modification of immune cells to maximize their therapeutic potential. CRISPR represents a significant leap forward in cancer immunotherapy by deactivating genes associated with immune inhibition, reducing the risk of GvHD and enhancing cytokine secretion. This offers promising prospects for improved treatment outcomes and prolonged survival rates in patients with complex cancers.

## 5. Enhancing Cancer Treatment: CRISPR/Cas9 Innovates Immunotherapy in Clinical Trials

Owing to the encouraging results from diverse and pioneering studies, numerous ongoing or completed clinical trials are utilizing CRISPR/Cas9 technology for cancer immunotherapy (Table 1). Notably, investigations specifically targeting PD-L1 in certain cancer types have not been documented. Instead, these clinical trials have predominantly focused on evaluating the safety, feasibility, and effectiveness of CRISPR-utilizing T cells. The delivery methods primarily involve non-viral approaches such as electroporation, microinjection, lipid nanoparticles (LNPs), and the use of a viral carrier utilizing a lentivirus [91,92,93,94,95]. These assessments, particularly Phases 1 and 2 clinical trials, were primarily in patients with hematologic malignancies or solid tumors and were conducted in the United States of America, China, and the United Kingdom. Phase I trials focused on assessing the safety of the treatment. As the trials progressed to Phase II, the scope broadened to include more participants and facilitated a comprehensive investigation of the effectiveness and safety profile. This phased approach plays an important role in ensuring that CRISPR-enabled cancer immunotherapy provides essential data before proceeding to extensive studies. The CRISPR technology plays a crucial role in enhancing the efficacy of T-cell therapy by targeting inhibitory molecules and signaling pathways in clinical trials. The simultaneous disruption of endogenous TCR, *β-2 B2M*, and PD-1 is strategically planned to create universal “off-the-shelf” CAR-T cells, aiming to reduce alloreactivity and GvHD [15,62,96]. PD-1 disruption, particularly in immunosuppressive environments, enhances CAR-T-cell effectiveness [77,97,98]. Genetic editing for creating allogeneic universal CAR-T cells provides accessible and improved therapies that enhance both potency and safety by integrating the CAR into the TCR locus [99]. Furthermore, CRISPR-mediated TGF-βR signaling disruption emerges as a powerful strategy to boost the activity of CAR-T cells against tumors [84,100]. The ongoing dedication to evolving and improving CRISPR-based cancer immunotherapy in these clinical trials signifies a commitment to explore innovative and effective strategies, ultimately aiming to enhance treatment outcomes for a broad spectrum of cancer patients. This relentless pursuit of innovation holds promise for revolutionizing cancer treatment paradigms and improving the lives of patients with various types of cancer.

As of December 2023, numerous ongoing clinical trials were using CRISPR/Cas9 for cancer immunotherapy. Table 1 shows a comprehensive list of ongoing and completed clinical trials registered on ClinicalTrials.gov, illustrating the diverse landscape of CRISPR/Cas9 applications in tumor immunotherapy.

## 6. Considerations and Strategies for CRISPR Delivery in T Cell Therapies

In the realm of CRISPR/Cas9-based T cell therapies, not only the introduction of CAR or TCR but also the delivery method for CRISPR components (Cas9 and gRNA) requires strategic consideration. Currently, in cases of CRISPR-based T cell therapies undergoing clinical trials, electroporation has been predominantly utilized for CRISPR delivery. When selecting a method to deliver CRISPR into T cells, several key factors must be considered: delivery efficiency, the maintenance of cell viability and function, safety, cost-effectiveness, time efficiency, and industrial scalability [7].

Delivery efficiency is crucial for effectively introducing the CRISPR/Cas9 system into T cells to perform desired genetic edits. Efficient delivery enhances therapeutic effects and accelerates treatment processes. Additionally, the delivery method should preserve cell viability and functionality, as any damage or functional impairment may compromise therapeutic efficacy. This consideration is particularly significant for adoptive cell transfer (ACT) therapies, where maintaining high cell viability during ex vivo T-cell expansion is challenging, emphasizing the importance of rapid and efficient delivery methods.

Safety considerations entail minimizing potential side effects of the delivery method on cells. Materials or techniques employed for intracellular delivery should exhibit minimal or no toxicity. Cost and time efficiency are vital for the commercialization of therapies. Cost-effective methods and swift delivery processes expand treatment accessibility to a broader population of patients. Industrial applicability necessitates selecting delivery methods suitable for large-scale production and commercialization, maximizing therapeutic benefits and widening patient access.

Regarding CRISPR types (DNA, mRNA, protein), several considerations arise. DNA-based CRISPR involves delivering the Cas9 gene and guide RNA (gRNA) together, ensuring stable maintenance and efficient expression within cells. However, additional verification processes to confirm potential off-target effects may incur time, cost, and affect production efficiency in the development of ex vivo manipulated cell therapies. mRNA-based CRISPR delivers Cas9 mRNA and gRNA to induce Cas9 protein expression within cells, necessitating the validation of mRNA stability and accurate translation.

Protein-based CRISPR delivers Cas9 protein and gRNA directly to cells externally, offering relatively straightforward solutions for overcoming nucleic acid-related challenges (such as off-target effects or expression errors), precise dosing predictability, and immediate functionality upon cell uptake. This format is considered optimal for immune cell targets where ex vivo culture is challenging. Comprehensive consideration of these factors is imperative for selecting an appropriate CRISPR delivery method. Although electroporation has been predominant in ongoing clinical trials for CRISPR delivery, alternative delivery methods are under research, and optimal selection should be based on individual advantages and disadvantages.

## 7. Navigating Safety Concerns in CRISPR Gene Editing for Clinical Cancer Immunotherapy

CRISPR is a game-changer in genetic engineering. However, the safety concerns when using CRISPR for gene editing in cells, particularly clinical applications such as cancer immunotherapy, should be carefully considered [5,12,101,102].

The potential of CRISPR lies in concerns regarding unintended genetic alterations. This central issue revolves around unintentional cleavage and mutations that can occur beyond the intended genetic target site. These off-target effects are contingent upon specific genetic sequences and induce oncogenic consequences [2,103,104,105].

CRISPR can initiate cell cycle arrest, trigger genomic instability, and increase harmful mutations via a p53-mediated DNA damage response, while also inadvertently inciting immune responses within T cells, which may lead to cytotoxicity. Additionally, incorrect chromosomal rearrangements, such as gene deletions and translocations, may be a potential outcome due to off-target interactions with genetic sequences [102,106].

The scientific community has made notable strides toward safer and more effective CRISPR-based therapies. Recent advancements have prioritized precise sgRNA design through programmability; engineering modified Cas9 variants, such as base or prime editors; and the development of sophisticated non-viral delivery methods, placing strong emphasis on safety and efficacy [26,46,47,107,108]. Furthermore, implementing spatiotemporal control technologies within the CRISPR system adds another layer of safety and accuracy to the process [67,102].

The optimization of various technical components in such CRISPR components significantly enhances both its specificity and functionality, improving the effectiveness of CRISPR while simultaneously minimizing off-target events [31,35,44,45,46]. Despite these remarkable advancements, there is still a need for sustained vigilance and ongoing research. In particular, a comprehensive understanding of the potential off-target mutagenesis-associated adverse effects is crucial to fulfill the promises of CRISPR-based therapies while maintaining the highest standards of safety and precision in the pursuit of innovative solutions for cancer immunotherapy [101,102,109].

## 8. Conclusions

The incorporation of CRISPR/Cas9 technology into cancer immunotherapy has enhanced the capacity to engineer precise and customized treatment strategies. CRISPR success lies in its unparalleled precision in target genetic sequence manipulation [1,27,31]. Guiding the Cas9 endonuclease with an sgRNA, CRISPR introduces changes with the utmost accuracy, propelling genetic engineering innovations across diverse cell types [2,35,36]. Its primary applications in cancer immunotherapy, particularly modulating the PD-1/PD-L1 pathway and engineering immune-specialized T cells, are pivotal for fine-tuning immunotherapy, amplifying T-cell activation, and circumventing the immune evasion strategies employed by cancer cells [17,23,85,110]. Targeting the PD-1/PD-L1 pathway not only fine-tunes immunotherapy, but also demonstrates promising potential in preclinical tests to overcome immune evasion strategies by cancer cells [9,62,98].

Furthermore, CRISPR-mediated genetic correction strategies for T cells range from modifying immune-specialized T cells to refining CAR-T-cell engineering [15,82,88,98,99]. The elimination of inhibitory genes like PD-1, *TGF-β*, and *PGE2*, coupled with the introduction of immune-boosting genes, indicates the versatility of CRISPR in maximizing the therapeutic potential of immune cells [64,84,100,111].

Several clinical trials have leveraged CRISPR technology for cancer immunotherapy, with a specific focus on evaluating the safety and efficacy of CRISPR-utilizing T cells. These trials emphasize the potential of universal “off-the-shelf” T cells, thus exploring innovative strategies and revolutionizing cancer treatment paradigms [9,82,89,98,112]. As CRISPR continues to advance, safety concerns are at a central stage. Consideration of off-target effects, unintended mutations, and potential adverse outcomes is crucial [101,102,109]. Noteworthy progress in sgRNA design, modified Cas9 variants, and sophisticated delivery methods has aimed to enhance safety and efficiency, highlighting the ongoing need for vigilance and research to ensure the highest precision standards [31,44,45,95,108]. In conclusion, CRISPR/Cas9 is a groundbreaking tool that not only refines genetic engineering, but also revolutionizes cancer immunotherapy. Its precision, adaptability, and application in clinical trials indicate its potential use in personalized and effective cancer treatment [22,25,113]. As research continues to unravel these complexities, CRISPR may transform our approach to combat cancer, offering innovative solutions to patients worldwide.

## Figures and Tables

**Table 1:** Clinical trials using CRISPR/Cas9 for cancer immunotherapy.

Clinical Trial Number	Phase	Cell	Edit Type	Drug	Target Gene	Delivery Methods	Delivery Cargos	Indication
NCT03545815	I	CAR-T	Knock-out	–	PD-1, TCR	Electroporation	RNP	Mesothelin-Positive Multiple Solid Tumors
NCT04037566	II	CAR-T	Knock-out	Cyclophosphamide, Fludarabine	*MAP4K1*	Electroporation	mRNA	Relapsed or Refractory Hematopoietic Malignancies
NCT03399448	I	NYCE T cells	Knock-in/knock-out	CTX/Fludarabine	TCRα, TCRβ, PD-1 knock-out, NY-ESO-1 knock-in	Electroporation	RNP	Multiple Myeloma Melanoma Synovial Sarcoma Myxoid/Round Cell Liposarcoma
NCT04244656	I	CAR-T	Knock-in/knock-out	CTX120	*B2M*, TCR	Electroporation	-	Multiple Myeloma
NCT04438083	I	CAR-T	Knock-in/knock-out	CTX130	*B2M*, TCR	Electroporation	-	Renal Cell Carcinoma
NCT04502446	I	CAR-T	Knock-in/knock-out	CTX130	*B2M*, TCR	Electroporation	-	T-Cell Lymphoma
NCT04035434	I/II	CAR-T	Knock-in/knock-out	CTX110	*B2M*, TCR	Electroporation	-	Relapsed or Refractory B-Cell Malignancies
NCT04637763	I	CAR-T	Knock-in/knock-out	CB-010	PD-1, TCR	–	-	Relapsed/refractory B-cell non-Hodgkin lymphoma
NCT04557436	I	CAR-T	Knock-in/knock-out	PBLTT52CAR19	*TRAC*, *CD52*	Electroporation, lentiviral vector (LV)	mRNA, sgRNA (viral)	B Acute Lymphoblastic Leukemia
NCT03747965	I	CAR-T	Knock-out	Paclitaxel, Cyclophosphamide	PD-1	–	-	Mesothelin-Positive Multiple Solid Tumors
NCT03166878	I/II	CAR-T	Knock-out	–	βTCRα, TCRβ, *β-2 B2M*	Electroporation	mRNA	Relapsed or Refractory CD19+ Leukemia and Lymphoma
NCT03081715	NA	T-Cells	Knock-out	–	PD-1	Microinjection	-	Advanced Esophageal Squamous Cell Carcinoma
NCT03398967	I/II	CAR-T	Knock-in/knock-out	–	*CD19*, *CD20*, *CD22*	Electroporation	RNP	Relapsed or Refractory Leukemia and Lymphoma
NCT04976218	I	CAR-T	Knock-out	–	*TGFBR2*	–	-	Advanced EGFR-positive Solid Tumors
NCT02793856	I	CAR-T	Knock-out	CTX	PD-1	Electroporation	Plasmid	Metastatic Non-small Cell Lung Cancer
NCT04767308	I	CAR-T	Knock-out	Cyclophosphamide, fludarabine	*CD5*	–	-	Relapsed/Refractory CD5+ Hematopoietic Malignancies
NCT04417764	I	T-Cells	Knock-out	–	PD-1	–	-	Advanced Hepatocellular Carcinoma
NCT03044743	I/II	EBV-CTL cells	Knock-out	Fludarabine, Cyclophosphamide, Interleukin-2	PD-1	Electroporation	Plasmid	Advanced Stage EBV Associated Malignancies
NCT05066165	I/II	WT1-TCR modified T cells	Knock-in/knock-out	NTLA-5001	TCRβ, TCRα knock-out, WT1-TCR integration	LNP	mRNA, sgRNA	Acute Myeloid Leukemia
NCT05397184	I	CAR-T	Knock-in/knock-out	BE CAR-7	*TRBC* *CD7*	Electroporation	mRNA	T-Cell Malignancies
NCT05643742	I/II	CAR-T	Knock-in/knock-out	CTX112	*TRAC*, *Regnase-1*, *TGFBR2*, *Β2M*	–	-	Relapsed or Refractory B-Cell Malignancies
NCT05812326	I/II	CAR-T	Knock-out	AJMUC-1	PD-1, *AJMUC1*	-	-	Advanced Breast Cancer Breast Neoplasm Malignant Female
NCT05566223	I/II	TILs	Knock-out	Fludarabine, Cyclophosphamide, Aldesleukin, Pembrolizumab	*CISH* (Cytokine-induced SH2 protein)	–	-	Metastatic Non-small Cell Lung Cancer

## Data Availability

No new data were created or analyzed in this study. Data sharing is not applicable to this study.
